# Effect of local versus remote tonic heat pain during training on acquisition and retention of a finger-tapping sequence task

**DOI:** 10.1007/s00221-015-4478-3

**Published:** 2015-11-02

**Authors:** Marie-Claude Bilodeau, Meyke Roosink, Catherine Mercier

**Affiliations:** Centre Interdisciplinaire de Recherche en Réadaptation et Intégration Sociale (CIRRIS), Quebec, QC Canada; Department of Rehabilitation, Faculty of Medicine, Laval University, Quebec, QC Canada

**Keywords:** Experimental pain, Cutaneous pain, Motor control, Motor performance, Learning

## Abstract

Although pain is present in a large proportion of patients receiving rehabilitation, its impact on motor learning is still unclear, especially in the case of neuropathic pain that is not tightly linked to specific movements. The aim of this study was to determine the effect of local and remote tonic cutaneous heat pain applied during training on motor learning of a finger-tapping sequence task. Forty-five healthy participants, randomized to the control, local pain or remote pain groups, were trained to perform an explicit finger motor sequence of five items as fast as possible. During the 10 training blocks (30 s each), local pain and remote pain groups received a heat pain stimulus on the wrist or leg, respectively. Performance was tested in the absence of pain in all groups before (baseline), immediately after (post-immediate), 60 min after (post-60 min) and 24 h after training (post-24 h) to assess both acquisition and next-day retention. Speed increased over time from baseline to post-24 h (*p* < 0.001), without any significant effect of group (*p* = 0.804) or time × group interaction (*p* = 0.385), indicating that the acquisition and retention were not affected by the presence of pain during training. No changes were observed on error rates, which were very low even at baseline. These results with experimental heat pain suggest that the ability to relearn finger sequence should not be affected by concomitant neuropathic pain in neurorehabilitation. However, these results need to be validated in the context of chronic pain, by including pain as a co-variable in motor rehabilitation trials.

## Introduction

It is well established that people move differently in the presence of pain. Motor performance (e.g., speed, accuracy) is affected, and adaptation to pain involves neuroplastic changes at multiple levels of the sensorimotor system (Bank et al. 2013; Hodges and Tucker 2011). The notion of a tight link between pain and movement is generally well accepted in the field of musculoskeletal pain (Boudreau et al. 2010; Hodges 2011; Hodges and Tucker 2011). Indeed, musculoskeletal pain is usually localized and triggered or exacerbated by specific movements. In contrast, the relationship between movement and neuropathic pain is less obvious. Neuropathic pain is one of the most common and disabling symptoms (besides motor deficits) that patients with neurological injury face (Flor 2002; Jonsson et al. 2006; Nakipoglu-Yuzer et al. 2013; Zanca et al. 2013), but is not necessarily localized or triggered by specific movements, nor necessarily spatially congruent with the location of sensorimotor deficits. Still, motor recovery has been reported to be inferior in patients with neurological injury with associated pain as compared to patients with similar injuries but without associated pain (Jonsson et al. 2006; Lundstrom et al. 2009; Roosink et al. 2011).

A better understanding of the relationship between pain localization (local, remote) and presentation (triggered by movement or not) and motor learning might be used to improve physical (neuro)rehabilitation outcomes in the presence of pain. As such, this experimental pain study aimed to determine the effect of local and remote pain presented during training on motor learning (i.e., acquisition and next-day retention), using a heat pain model and a finger-tapping sequence task. The rationale for this approach is presented below.

A cutaneous (thermode-induced) heat pain model was selected for two main reasons: (1) burning pain mimics neuropathic pain; (2) compared to capsaicin, which has often been used in previous studies on the interaction between pain and motor learning (Boudreau et al. 2007; Bouffard et al. 2014; Dancey et al. 2014; Lamothe et al. 2014), it allows the measurement of performance before and right after training in the absence of pain (disentangling the effect of pain on motor learning from the immediate effect of pain on motor performance during assessment). Cutaneous heat pain provides a model in which pain intensity is not aggravated by the trained movement itself. This is important given that in the only previous study reporting decreased motor acquisition during training, pain was directly modulated by the trained task itself since participants were required to push against a lever with their tongue that was sensitized with capsaicin (Boudreau et al. 2007). In contrast, others reported either similar (Bouffard et al. 2014; Ingham et al. 2011; Lamothe et al. 2014) or even increased (Dancey et al. 2014) performance when training was realized in the presence (vs. the absence) of capsaicin-induced pain that was not modulated by the task itself. Notably, in these studies, pain was generally localized on the body segment directly involved in the trained motor task (Boudreau et al. 2007; Bouffard et al. 2014; Lamothe et al. 2014). To date, only one study assessed the effect of both local and remote (intramuscular) pain and proposed that remote pain may compromise learning due to distraction from the training task or from other complex central pain processes while local pain does not (Ingham et al. 2011). Indeed, other studies have proposed the existence of a bidirectional relationship between pain and motor task performance that engage overlapping cognitive resources (Buhle and Wager 2010; Legrain et al. 2009). As such, it is of interest to investigate further whether local and remote pain have similar effects as pain location do not always coincide with motor deficits location in patients with injuries to the nervous system.

A finger-tapping sequence task was selected for several reasons. First, only one of the previous studies investigated the impact of pain on the performance of a motor task involving manual dexterity (Dancey et al. 2014), which is surprising given the fact that regaining manual dexterity is an important rehabilitation goal in several populations with neurological injuries and that most neurophysiological studies on the impact of pain on corticospinal excitability have focused on hand muscles (Cheong et al. 2003; Farina et al. 2001; Fierro et al. 2010; Kaneko et al. 1998; Kofler et al. 1998, 2001; Le Pera et al. 2001; Urban et al. 2004; Valeriani et al. 1999, 2001). Second, this type of task is particularly suitable for studying retention, and spontaneous improvement in performance can even be observed between practice sessions, without any further training, a phenomenon called off-line improvement (Doyon et al. 2009a; Robertson et al. 2004). Importantly, factors improving or decreasing short-term motor acquisition are not always predictive of motor retention and as such it is crucial to investigate retention in order to truly characterize motor learning (Kantak and Winstein 2012). Only two recent studies investigated the impact of pain (capsaicin model) on motor retention so far, using motor adaptation tasks (force field) during a locomotor or an upper limb reaching task (Bouffard et al. 2014; Lamothe et al. 2014). For the locomotor task, it was shown that the presence of pain during task acquisition left task acquisition unaffected but reduced task retention (Bouffard et al. 2014), whereas for the reaching task, the presence of pain during task acquisition impacted on movement strategies (but not on performance) both during task acquisition and task retention (Lamothe et al. 2014). Studying a motor sequence learning task, that relies on partially independent brain networks than those involved in motor adaptation (Doyon and Benali 2005), will provide complementary information.

## Experimental procedures

### Participants

Recruitment was done through an advertisement at the local university. Forty-five healthy subjects participated in the study. They were all right-handed according to the Edinburgh Handedness Inventory (Oldfield 1971). Individuals that played a musical instrument on a regular basis (≥1×/week) during the last 3 years were excluded from participation to avoid too much familiarity with sequential manual tasks. Additional exclusion criteria were acute or chronic pain of any kind, a history of neurological or psychiatric disorders, or musculoskeletal disorders involving the left hand.

### Finger-tapping task

**Fig. 1:**
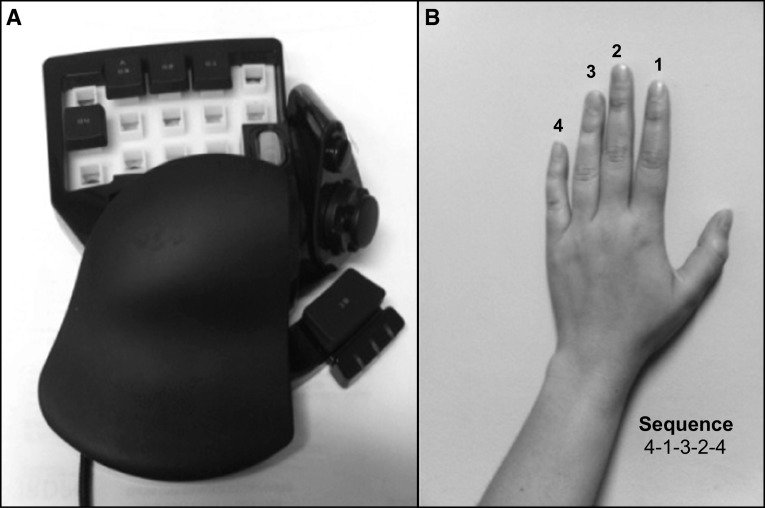
**a** Response box “Razer Nostromo Gaming Keypad,” **b** sequential finger-tapping task

Participants were trained to perform a finger-tapping task of five items on a response box on which four keys were arranged ergonomically for the left hand (see Fig. 1a). The task required reproducing the sequence 4-1-3-2-4 with the non-dominant hand as quickly and accurately as possible for a period of 30 s (where 1 corresponds to the index and 4 to the little finger, see Fig. 1b). The numeric sequence was continuously displayed on a computer screen to reduce the demands on working memory to a minimum (Albouy et al. 2013; Walker et al. 2002). No feedback on the accuracy of performance was provided: upon each key press, a gray dot appeared on the screen forming a row from left to right. Instruction was given that occasional errors should not be corrected, and participants had to continue the task without pause.

### Design

Experiments were carried out on two consecutive days to evaluate motor acquisition (Day 1) and next-day retention (Day 2). For a given subject, the two sessions were held at the same time of the day (see Table 1 for experimental design).

**Table 1:** Experimental design

Day 1							Day 2
Familiarization	Baseline evaluation	Training with/without pain	Post-immediate evaluation	Pause	Post-60 min evaluation	Pause	Post-24 h evaluation
Finger position Video Verify sequence	2 × 30 s	10 × 30 s	2 × 30 s	1 h	2 × 30 s	24 h	2 × 30 s

Experiments were carried out on two consecutive days to evaluate motor acquisition (Day 1) and next-day retention (Day 2)

#### Day 1

On Day 1, participants were first familiarized with the response box. After positioning their hand on the response box, the sequence (performed slowly by a hand model) was presented 4 times on video. Familiarization was considered successful when participants were able to repeat the sequence at least three times without errors. This was followed by a baseline evaluation of task performance (baseline), a training session, and a post-training evaluation of task performance (post-immediate and post-60 min). Evaluation of task performance consisted of performing the task during two blocks of 30 s. During training, the task was performed for 10 blocks of 30 s. Both during evaluations and training, blocks were interspersed with 30-s rest periods during which the computer screen turned white. An auditory signal 5 s before the start notified the participant to get prepared for the next block.

In addition, computer use (hours/week) was recorded.

#### Day 2

On Day 2 (24 h after the training session), a sleep diary of the previous night was completed followed by a last post-training evaluation of task performance (post-24 h) (2 × 30 s).

#### Experimental pain during training

Participants were randomized to the control group (no experimental pain), the local pain group or the remote pain group (10 women and five men in each group). An experimenter-operated 3 × 3 cm thermode (Model TSA-II, Medoc Advanced Medical Systems, Durham, U.S.) was used to produce pain by applying a hot nociceptive stimulus. For the local pain group, pain was applied to the dorsal face of the left wrist (the hand performing the task). For the remote pain group, pain was applied to the external face of the left leg, just below the knee. The experimenter was sitting next to the participant throughout the experiment regardless of group assignment.

At the very beginning of the experiment, for all participants (including the control group) the temperature of the thermode was individually adjusted to reach a target pain level that was rated as 4–5 according to a numeral rating scale (NRS) of 0 (no pain) to 10 (worst pain imaginable). A first test was done at 47 °C for the wrist or at 46 °C for the leg for a period of 30 s. Then, the temperature was increased or decreased in steps of 1 °C to reach the target pain level. Group assignment was revealed to the participant only after the baseline evaluation, just before the training. For the local and remote pain groups, experimental pain was present only during training on Day 1 and not during any of the evaluations on Day 1 or Day 2. The stimulation temperature was kept constant during the training blocks and was interrupted during the rest periods. Pain was monitored after each training block using an NRS.

### Primary outcome parameters

The following two variables were considered: (1) *error rate* (mean number of errors per completed sequence, reflecting accuracy) and (2) *speed* (number of completed sequences per 30 s).

For each evaluation time point, the two evaluation blocks were considered as associated pairs and the block corresponding to the best performance (depending on the number of correctly completed sequences per 30 s) was used for analysis.

### Statistical analyses

To compare the characteristics of participants between groups at baseline, one-way ANOVAs or Student’s *t* tests were performed. Two-way repeated measures analyses of variance (ANOVA) were performed to assess the effect of group (control, local pain and remote pain) and time (baseline, post-immediate, post-60 min and post-24 h) for the two dependent variables (*error rate* and *speed*) separately. A two-way ANOVA (group × time) was also performed to assess the evolution of pain across training blocks. When the assumption of homogeneity was violated (based on Mauchly’s test of sphericity), the Greenhouse–Geisser correction was applied. Post hoc analyses were performed using a Sidak correction for multiple comparisons. A *p* value <0.05 was considered significant.

## Results

### Participants’ characteristics

**Fig. 2:**
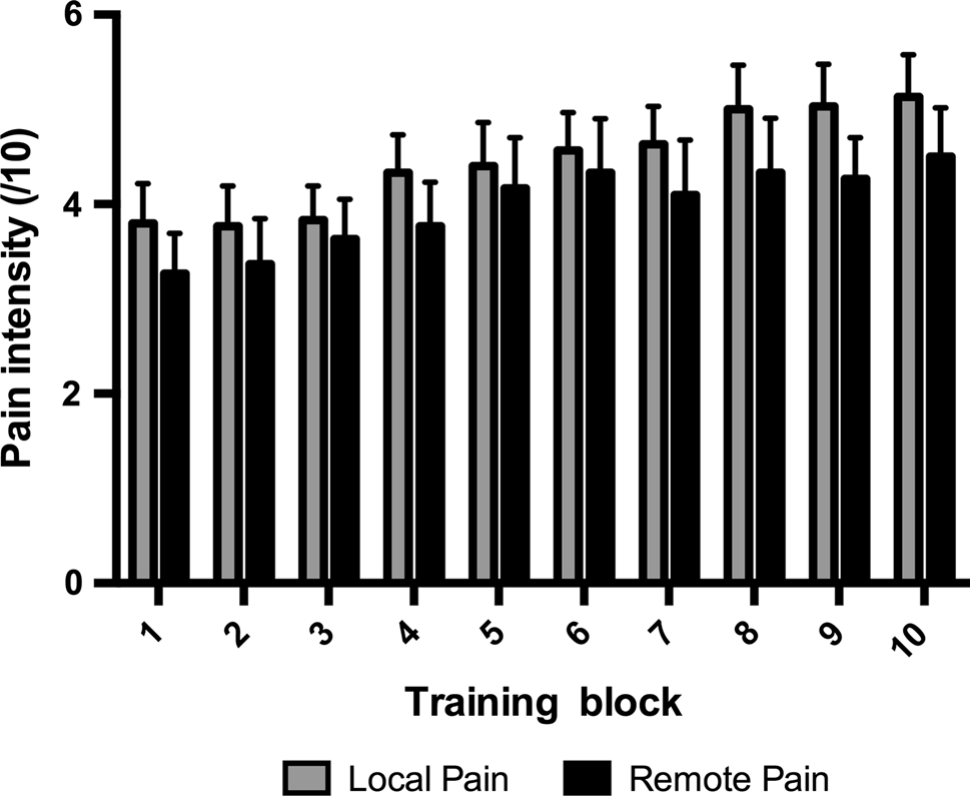
Average perceived pain intensity (/10) across the 10 training blocks as a function of group (for local and remote pain group only, as no pain was reported in the control group). Data are presented as mean ± SE of the mean

Forty-five subjects participated in the study (see Table 2). Groups did not statistically differ with respect to age (*p* = 0.896), computer use (*p* = 0.485) or hours of sleep between Day 1 and Day 2 (*p* = 0.124). At baseline, no significant differences were found when comparing the three groups according to *Error Rate* (*F*_(2,42)_ = 0.170, *p* = 0.844) and *Speed* (*F*_(2,42)_ = 0.391, *p* = 0.679). During the training, all groups had equal amounts of practice, i.e., the total number of completed sequences during the ten blocks was not significantly different between groups (*p* = 0.648). Figure 2 shows the evolution of the perceived pain intensity across the 10 training blocks. A significant increase in perceived intensity was observed over time (*F*_(9,252)_ = 7.734, *p* < 0.001), with an average rise of 1.2/10 on the NRS from the first to the last block of training. No effect of group (*F*_(1,28)_ = 7.734, *p* = 0.414) or time by group interaction (*F*_(9,252)_ = 0.351, *p* = 0.851) was observed. This indicates that perceived pain intensity was successfully matched across local and remote pain groups, although requiring slightly different stimulation temperatures (*p* = 0.013).

**Table 2:** Participants’ characteristics

	Control group	Local pain group	Remote pain group
*N* (women/men)	15 (10:5)	15 (10:5)	15 (10:5)
Age (years)	28.8 ± 8.8	27.4 ± 7.1	28.5 ± 9.5
Use of computer (hours/week)	29.9 ± 14.6	28.3 ± 17.4	22.8 ± 18.1
Sleep between Day 1 and Day 2 (hours)	6.9 ± 1.4	7.0 ± 1.4	7.8 ± 1.0
NRS rating during training (0–10)		4.5 ± 1.3	3.9 ± 1.8
Temperature of stimulus during training (°C)		46.8 ± 0.7	46.1 ± 0.8
Practice during training (number of completed sequences)	191.1 ± 20.5	177.1 ± 12.9	197.2 ± 11.9

Data are presented as number of participants or as mean ± SD

### Error rate

**Fig. 3:**
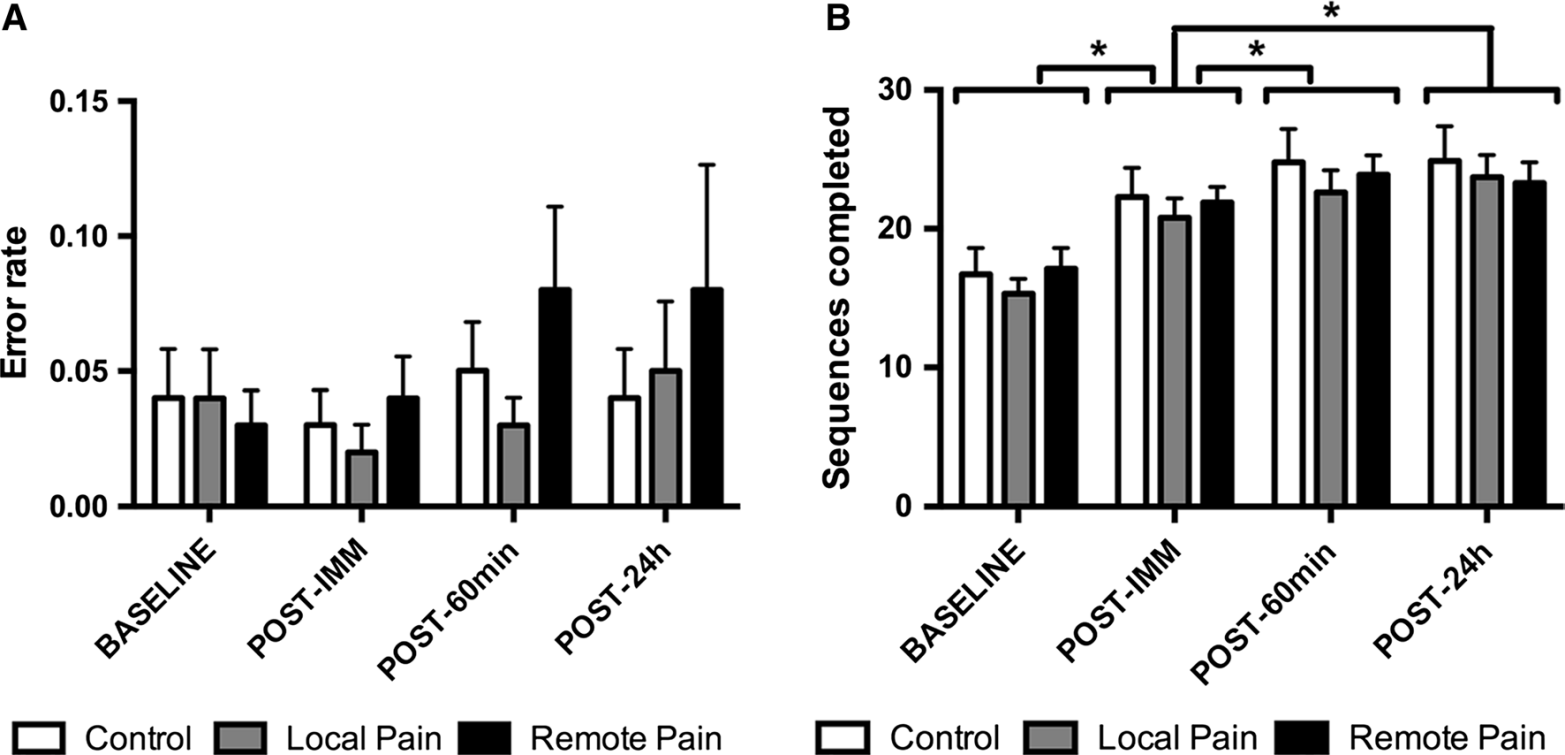
**a** Average *error rate* (mean number of errors per completed sequence) and **b** *speed* (number of completed sequences per 30 s) as a function of group and time. Data are presented as mean ± SE of the mean. *p < 0.001

*Error rates* were found to be very low, as shown in Fig. 3. On average, subjects performed 97.1 ± 5.0 % of the sequences correctly. Consequently, no overall effect of time (*F*_(3,40)_ = 2.468, *p* = 0.076), no effect of group (*F*_(2,42)_ = 1.296, *p* = 0.284) and no interaction of time by group (*F*_(6,82)_ = 0.700, *p* = 0.650) were observed.

### Speed

Data for *speed* are presented in Fig. 3. An overall effect of time was seen across the different time points of evaluation (baseline, post-immediate, post-60 m and post-24 h), (*F*_(3,40)_ = 92.881, *p* < 0.001), indicating that, overall, *speed* increased over time from baseline to post-24 h. Post hoc contrasts showed that this increase in *speed* was significant when comparing baseline and post-immediate to all other time points (for all *p* < 0.001), but not when comparing post-60 m to post-24 h (*p* = 0.988). No effect of group (*F*_(2,42)_ = 0.220, *p* = 0.804) nor time × group interaction (*F*_(6,82)_ = 1.074, *p* = 0.385) was observed, indicating that motor acquisition and next-day retention were not affected by the presence of tonic heat pain during training.

## Discussion

The aim of this study was to determine the effect of acute local and remote experimental tonic heat pain presented during training on the acquisition and next-day retention of a finger-tapping sequence task. Regardless of the presence of pain, the *speed* increased from pre- to post-training, while the *error rate* was constant over time. As such, it seems that local and remote tonic heat pain did not affect the acquisition and/or the next-day retention of the finger-tapping sequence task.

It appears unlikely that this absence of interference with learning would be due to the experimental design. For example, when looking at the motor task, the *speed* observed at baseline (~16 sequences) was comparable to that reported in the literature using this type of task (Doyon et al. 2009b; Karni et al. 1995; Walker et al. 2002, 2003), and a clear improvement of performance was observed over time, also comparable in magnitude to that of previous studies (Doyon et al. 2009b; Walker et al. 2002, 2003). However, the error rates were very low, presumably because the numeric sequence was continuously displayed, and it is possible that a more complex task involving a stronger cognitive component would be more susceptible to interference from pain. Pain level during training was also close to the targeted pain level (mean of 4.5/10 for the local pain group and 3.9/10 for the remote pain group), and subjects did not adapt to tonic heat pain during training (conversely, a gradual increase in pain was reported over time). These pain levels are comparable to those of previous studies showing an impact of pain on motor learning (Boudreau et al. 2007; Bouffard et al. 2014; Dancey et al. 2014). Moreover, a study looking at the effect of pain on M1 excitability showed that even a low-to-moderate level of heat pain (intensity of 2.8 ± 1.9/10) is sufficient to inhibit corticospinal excitability, suggesting that such a pain level might be sufficient to interfere with motor learning (Dube and Mercier 2011). The sample size was also comparable to that of previous studies that have shown a significant effect on motor learning (Bouffard et al. 2014; Dancey et al. 2014; Lamothe et al. 2014).

Then, how can we explain the discrepancies in the effect of pain on motor learning reported across studies? A new theory was recently proposed to explain movement adaptation to pain, highlighting the fact that pain can have effects at multiple sites along the motor pathways which may be complementary, additive or competitive (Hodges and Tucker 2011). Because of that, the net effect of pain the motor system is likely to vary both within (i.e., depending on effectors, types of motor tasks, and types of pain) and between individuals, which may account for some of the variability in experimental findings. Given that performance was not found to be more variable across individuals in the pain groups compared to the control group within our study (see Fig. 3), between-subject variability is unlikely to explain the negative findings. Other factors that might contribute to explain discrepancies between our results and that of previous studies are outlined below. Importantly, although these factors are discussed independently for clarity reasons, it is likely that a combination of these factors account for differences between studies, with potential interactions between factors (i.e., a specific type of pain might interfere more with a specific type of motor task).

Regarding the effector, the two studies that have shown a negative impact of pain on motor learning involved either the tongue (protrusion movements) (Boudreau et al. 2007) or the leg (gait) (Bouffard et al. 2014), while studies focusing on upper limb have reported either no effect or a positive effect on motor performance in response to motor training (Dancey et al. 2014; Ingham et al. 2011; Lamothe et al. 2014), consistent with the results of the present study (although Ingham et al. 2011 reported reduced cortical plasticity in the presence of remote pain). It could be hypothesized that movements relying more on subcortical control could be more vulnerable to pain interference. Animal studies showing that brain-dependent mechanisms can play a protective role against spinal learning deficits induced by nociceptive stimuli provide some indirect evidence supporting that view (Grau et al. 2014). However, this remains speculative given both force field adaptations during gait and force tracking with the tongue are likely to depend both on cortical and subcortical (i.e., spinal or brainstem) structures (Barthelemy et al. 2012; Sawczuk and Mosier 2001).

Regarding the type of task, it has been suggested that sequence motor learning and force field adaptation tasks rely on different brain mechanisms, the former depending more on cortico-striatal plasticity and the latter more on cortico-cerebellar plasticity (Doyon and Benali 2005). Hemodynamic changes in the cerebellum and striatum have been reported during pain in several neuroimaging studies (in addition to M1 and SMA) (Apkarian et al. 2005; Farina et al. 2003; Peyron et al. 2000). This raises the possibility that pain could interfere with learning relying on cortico-striatal plasticity, cortico-cerebellar plasticity or both. However, the effect of pain on each of these two pathways could be different, as the physiological impact of these hemodynamic changes is still unclear.

Finally, different types of pain could also have a different impact on motor acquisition and retention. For example, in one of the studies showing an impact of pain on motor acquisition, participants were required to push against a lever with their tongue that was sensitized with capsaicin. Thus, task performance provoked movement-related pain in the task effector, which might have impacted on the amount of practice during training (e.g., lower force amplitude or duration). This contrasts with the type of pain that was used in the present and in other studies (Bouffard et al. 2014; Dancey et al. 2014; Lamothe et al. 2014), which was not directly related to the trained movement. Notably, while Bouffard et al. 2014 reported an effect of constant pain induced by capsaicin on *retention* of motor learning, no effect was observed on *motor acquisition* during training. Another potentially important aspect is that in the present study all assessments of performance were done in the absence of pain. This contrasts with studies using capsaicin, in which motor performance at the end of training (i.e., motor acquisition) is measured while the subject is still in pain.

The absence of effect of local (wrist) or distant (leg) tonic heat pain in the present study [as well as in the previous study on the effect of capsaicin-induced pain on a manual sequence task (Dancey et al. 2014)] suggests that the ability to relearn this type of task should not be affected by the presence of concomitant neuropathic pain in patients with motor deficits affecting the hand. However, the impact of chronic pain could differ from that of acute pain in several ways. Chronic pain is generally more severe and affects a larger territory than the heat pain model that was used in the present study. Moreover, sensitization occurs in response to injury or sustained nociceptive input (Rahn et al. 2013). Individuals with chronic pain also often exhibit pain-related fear leading to avoidance of movement and activity (Zale et al. 2013) that can affect motor training and learning. Finally, factors such as the use of medication, sleep problems, etc. could also interfere with motor acquisition or consolidation (Doyon et al. 2009b; Hook et al. 2007). Therefore, it would be needed to validate these results in a clinical population. This could be achieved by assessing pain as a co-variable in future clinical trials targeting manual dexterity, something that is typically not done in this type of study.

## Conclusion

The current study showed that neither local nor remote acute tonic heat pain interfered with motor learning of a sequence finger-tapping task. Further research is needed to clarify the discrepancies observed between studies that have used different effectors, types of motor task and types of pain. This will help clarify if and how rehabilitation interventions involving motor learning need to be adapted in patients presenting motor deficits with concomitant pain.
